# Bone mineral loss induced by anticancer treatment for gynecological malignancies in premenopausal women

**DOI:** 10.1530/EC-12-0043

**Published:** 2012-11-19

**Authors:** Keina Nishio, Akiko Tanabe, Risa Maruoka, Kiyoko Nakamura, Masaaki Takai, Tatsuharu Sekijima, Satoshi Tunetoh, Yoshito Terai, Masahide Ohmichi

**Affiliations:** 1 Department of Obstetrics and Gynecology Osaka Medical College 2-7 Daigaku-machi, Takatsuki-city, Osaka, 569-8686 Japan

**Keywords:** surgical menopause, concurrent chemo-radiation therapy, bone mineral density, cervical cancer

## Abstract

**Objective:**

Although surgical menopause may increase the risks of osteoporosis, few studies have investigated the influence of chemotherapy and radiation therapy. The aim of this study is to evaluate the effects of treatments for gynecological malignancies on bone mineral density (BMD).

**Methods:**

This study enrolled 35 premenopausal women (15 ovarian cancers (OCs), 9 endometrial cancers (ECs), and 11 cervical cancers (CCs)) who underwent surgical treatment that included bilateral oophorectomy with or without adjuvant platinum-based chemotherapy in OC and EC patients, or concurrent chemo-radiation therapy (CCRT) in CC patients according to the established protocols at the Osaka Medical College Hospital between 2006 and 2008. The BMD of the lumbar spine (L1–L4) was measured by dual-energy X-ray absorptiometry, and urine cross-linked telopeptides of type I collagen (NTx) and bone alkaline phosphatase (BAP) were assessed for evaluation of bone resorption and bone formation respectively. These assessments were performed at baseline and 12 months after treatment.

**Results:**

Although the serum BAP was significantly increased only in the CC group, a rapid increase in the bone resorption marker urinary NTx was observed in all groups. The BMD, 12 months after CCRT was significantly decreased in the CC group at 91.9±5.9% (*P*<0.05 in comparison to the baseline).

**Conclusion:**

This research suggests that anticancer therapies for premenopausal women with gynecological malignancies increase bone resorption and may reduce BMD, particularly in CC patients who have received CCRT. Therefore, gynecologic cancer survivors should be educated about these potential risks and complications.

## Introduction

Cancer survivors are living longer after the initial diagnosis of cancer due to earlier diagnosis and improved cancer treatments. Epithelial ovarian cancer (OC), uterine endometrial cancer (EC), and cervical cancer (CC) are major gynecological malignancies. Patients with these diseases generally undergo surgery that includes a bilateral oophorectomy followed by the administration of platinum-based chemotherapy in OC and EC patients, or concurrent chemo-radiation therapy (CCRT) in CC patients. Although, the acute side effects of combination chemotherapy and CCRT are well defined, the late adverse effects have not been considered after adjuvant anticancer treatments. The long-term toxicity of surgical menopause and anticancer chemotherapies are important because these patients have an additional expected life span.

Numerous older studies have shown that bone loss either starts or increases at the time of the menopause (1, 2, 3). The earlier in life that menopause occurs, the lower the bone density will be later in life (4). Ohta *et al*. (5) compared age-matched premenopausal women at age 49 years with oophorectomized women 6 years past menopause. The authors showed that bone loss measured by quantitative computerized tomography was significantly higher in comparison to natural menopause women. In addition, risk of osteoporotic fracture may be increased in women who undergo bilateral oophorectomy after natural menopause in comparison to women with intact ovaries (6, 7).

Osteoporosis is common in women with breast cancer who receive chemotherapy, hormone therapy, or surgical castration, because these treatments induce bone loss (8). Atkinson *et al*. (9) reported that prepubertal children with lymphoblastic leukemia who received anticancer agents also suffered bone mineral loss. It is likely that, in addition to hypogonadism, other factors induce bone mineral loss. Bone mineral loss during chemotherapy has been shown to be greater than that following castration alone (10). Accelerated bone loss may occur through the direct effects of chemotherapy on bone, because methotrexate and ifosfamide are suggested to have direct bone effects (11, 12). However, most chemotherapy in clinical practice is given as a combination of several agents and chemotherapy is often combined with steroid therapy, which will contribute to bone loss.

High dose local radiation therapy, is associated with atrophy of the trabeculae of bone, i.e. osteoporosis, particularly after kilovoltage irradiation (13). The number of osteoblast cells is reduced after irradiation, and this phenomenon is associated with a decreased collagen production and alkaline phosphatase activity (14). Changes in the blood flow have also been reported in bone after irradiation in the femur of rats, following bone atrophy (15). However, the radiation therapy-related bone loss following the treatment of gynecological malignancies remains controversial. Therefore, this study investigated premenopausal women with gynecological malignancies that received surgical castration followed by adjuvant chemotherapy or CCRT, and evaluated the effects of these treatments on bone density.

## Materials and methods

### Patients

This study was approved by the institutional review board of Osaka Medical College. The study population included 35 women (15 with OCs, nine with ECs, and 11 CCs), ranging from 32 to 49 years of age with regular menstruation, who underwent surgical treatment with adjuvant chemotherapy or CCRT according to the established protocols from January 2006 to December 2008. Women with a current or past medical condition or medication use that may influence bone mineral metabolism (hyperthyroidism, rheumatoid arthritis, history of fractures that can be explained by trauma such as those occurring during motor-vehicle accidents, corticosteroid drug use, hormone replacement therapy, use of oral contraceptives for a period longer that 6 months, past or recent use of hormone therapy) were excluded from this study. In addition, subjects with a BMI higher than 40 kg/m^2^ were excluded, due to the artifacts in bone mineral density (BMD) measurements with the dual-energy X-ray absorptiometry (DXA) unit used.

### Treatment design

#### Ovarian cancer

Patients with invasive epithelial OC underwent a total hysterectomy, bilateral salpingo-oophorectomy, pelvic/para-aortic lymphadenectomy, and omentectomy including complete removal of all visible tumor tissue. Paclitaxel plus carboplatin (TC) chemotherapy was administered to all patients (surgery+TC group) except for the low risk according to established protocols, after staging classification using the International Federation of Gynecology and Obstetrics (FIGO) nomenclature. Briefly, women recommended to undergo chemotherapy were premedicated with dexamethasone (20 mg i.v.) for 30 min before the start of paclitaxel infusion. Both diphenhydramine (50 mg orally) and ranitidine (50 mg i.v.) were also administered 30 min before paclitaxel infusion. Next, the patients received paclitaxel (175 mg/m^2^) i.v. for 3 h, followed by carboplatin (AUC 5 mg/ml) i.v. for 1 h on day 1 every 3 weeks for a total of six courses. The carboplatin dose was calculated using the Calvert formula; carboplatin dose (in mg)=AUC×(GFR+25). The glomerular filtration rate was estimated using the Jelliffe formula. A total hysterectomy, bilateral salpingo-oophorectomy, and omentectomy were performed in patients with borderline malignant epithelial tumors.

#### Endometrial cancer

All patients underwent a total hysterectomy and bilateral salpingo-oophorectomy, followed by the observation of an intraoperative frozen section. A pelvic/para-aortic lymphadenectomy was performed in patients with more than intermediate risks according to established protocols. TC chemotherapy was performed in all patients for a total of three courses (surgery+TC group).

#### Cervical cancer

CC was confirmed by colposcopic directed biopsies. The preoperative workup included magnetic resonance imaging of the pelvis and CT scan of the lower abdomen in all women. Stage Ia disease with low risk factors, stages III and IV disease were excluded in this study according to the FIGO staging classification. All patients underwent a radical hysterectomy, bilateral salpingo-oophorectomy, and a pelvic lymphadenectomy, followed by CCRT. Pelvic radiotherapy was delivered using a 10 MV X-ray from a linear accelerator with the anteroposterior parallel opposing technique. The superior margin of the external radiation field was placed on the upper border of the fifth lumber vertebra, and the inferior margins were the inferior border of the obturator foramen. The field extended 2 cm beyond the lateral margin of the bony pelvic wall. Multi-leaf collimators were used to block the upper and lower corners of the radiation field. External irradiation was delivered to the whole pelvis at 2 Gy per fraction for 5 fractions per week, for a total of 25 fractions (50 Gy). Cisplatin was given intravenously at 40 mg/m^2^ on a weekly basis during the course of EBRT for 5 weeks. The drug was given in a 1 h infusion. In addition, the renal function and blood counts were assessed before each cycle.

### Study design

**Figure 1:**
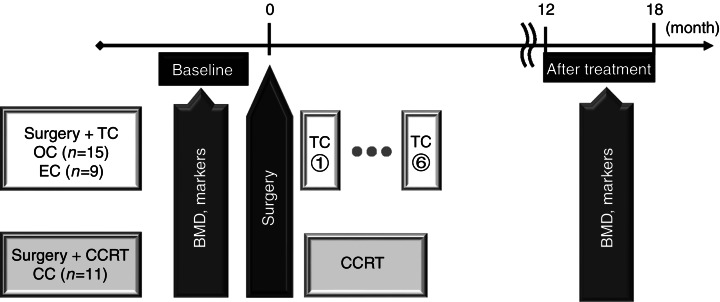
Study design. TC, paclitaxel plus carboplatin chemotherapy; OC, ovarian cancer; EC, endometrial cancer; CC, cervical cancer; BMD, bone mineral density; CCRT, concurrent chemo-radiation therapy.

The study design is shown in Fig. 1. Bone mineral densities and bone markers were measured before surgery (baseline) as well as at 12 months after treatment. Bone densities of four lumbar vertebrae (L1, L2, L3, and L4) were measured by DXA using iDXA/PRODIGY (GE Healthcare, Buckinghamshire, UK), and reported as grams per centimeter squared. The mean BMD was calculated using the scanner software. *T*-score was calculated using the National Health and Nutrition Examination Survey III (NHANESIII) database. The serum bone-specific alkaline phosphatase (BAP) level was measured on fasting samples as a bone formation marker. The reference range was 9.6 to 22.6 U/l. Bone resorption was assessed by measurements of the urinary amino-terminal telopeptide fragment of type I collagen (NTx) in the early morning using a second voided urine sample which was collected on the day of outpatient attendance. The NTX reference range for premenopausal women was 9.3–54.3 nmol bone collagen equivalent (BCE)/mmolCr.

### Statistical analyses

All of the data in the Tables and Figures are expressed as the mean±s.d. The Kolmogorov–Smirnov test was used to determine whether continuous variables were normally distributed. ANOVA was used for group comparison. The values were compared using Wilcoxon's test as a non-parametric test to analyze differences between the values recorded at baseline and at 12 months after the start of the study. A value of *P*<0.05 was considered to be statistically significant.

## Results

### Baseline values of clinical variables

The clinical characteristics of the participants are presented in Table 1. There were no significant differences in the age, BMI, bone turnover markers, and BMD between the cancer types at baseline.

### Effect of anticancer therapies on bone turnover markers in premenopausal patients

**Figure 2:**
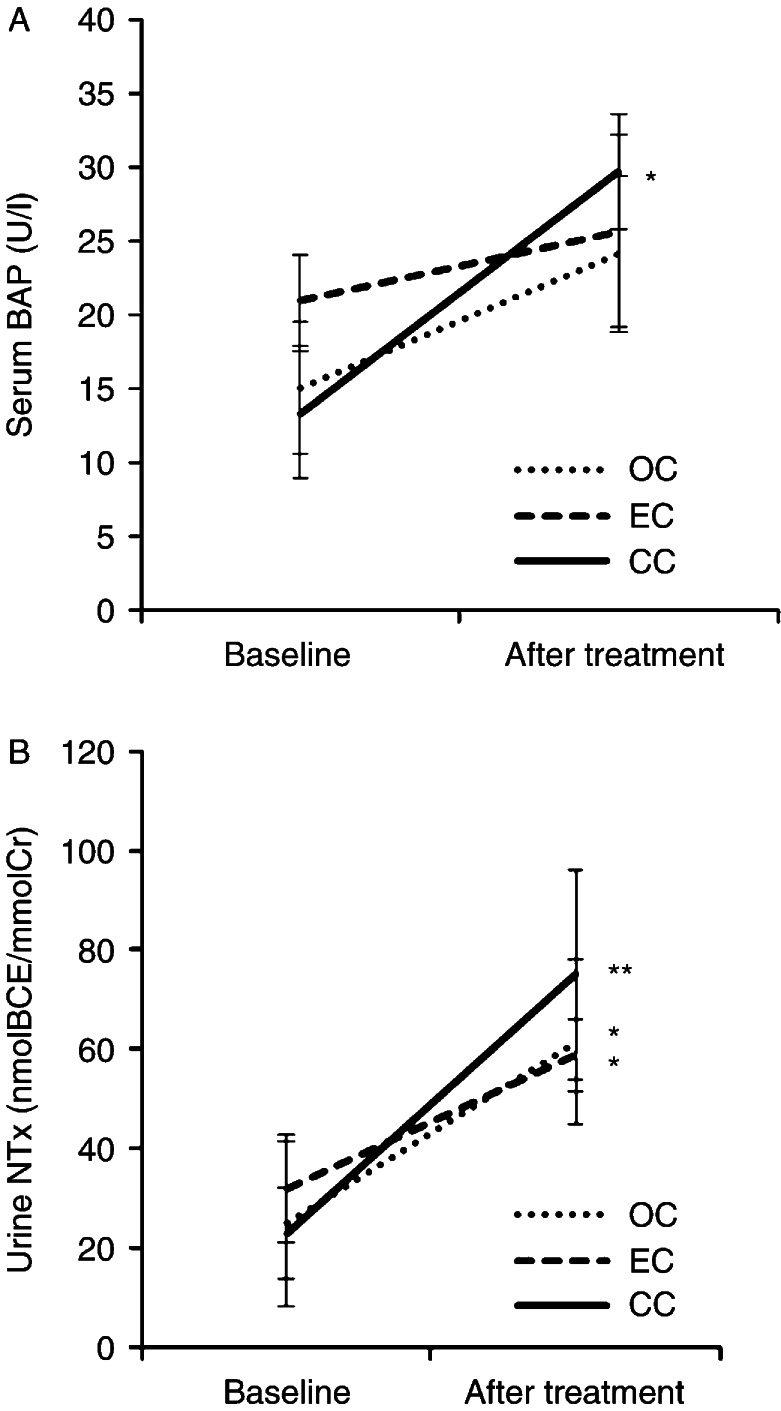
Effect of anticancer treatment on bone turnover markers. Serum BAP (A) and urine NTx (B) were measured before surgical treatment (baseline) and at 12 months after the cancer treatment (after treatment). Vertical bars represent the mean±s.d. A value of *P* is shown in comparison to the baseline. Significant differences are indicated by asterisks. *P<0.05; **P<0.01.

Bone turnover markers were measured before surgery (baseline) and at 12 months after anticancer treatment in the premenopausal patients (Fig. 2A and B). The OC and EC groups showed slightly but not significantly elevated serum BAP, from 15.1±4.5 to 24.2±5.3 U/l and 21.0±3.1 to 25.7±6.5 U/l respectively. In other hand, CC groups who were treated with CCRT after surgical treatment showed a significantly elevated serum BAP level from 13.3±4.3 to 29.8±3.9 U/l (*P*<0.05; Fig. 2A). Urine NTx was significantly elevated from 25.0±9.2 to 61.5±21.1 U/l (*P*<0.05) in the OC group, from 32.0±1.9 to 58.8±7.3 U/l (*P*<0.05) in the EC group, and from 23.1±16.6 to 75.1±16.5 U/l (*P*<0.01) in the CC group after anticancer treatments.

### Effect of anticancer therapies on lumbar spine BMD in premenopausal patients

**Figure 3:**
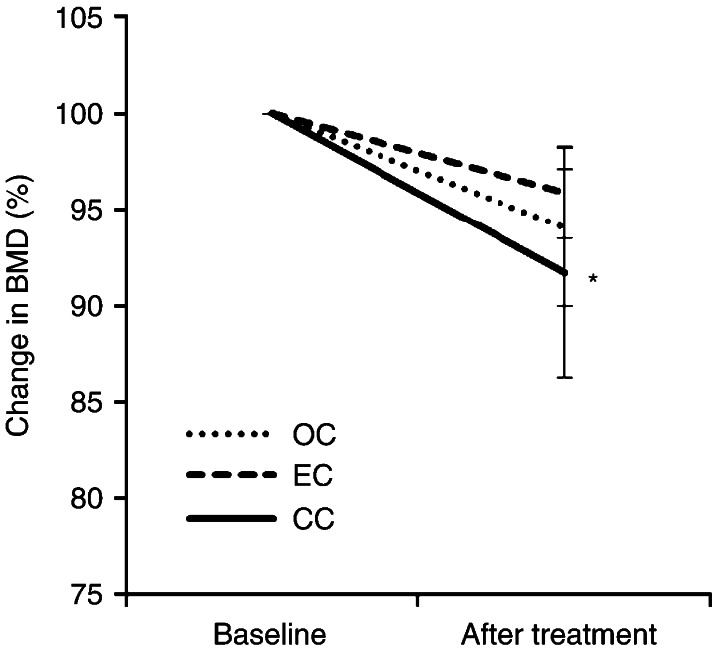
Effect of anticancer treatment on bone mineral density. The lumbar spine BMD was measured before surgical treatment (baseline) and at 12 months after the cancer treatment (after treatment). The percentage change from baseline is shown. Vertical bars represent the mean±s.d. A value of *P* is shown in comparison to the baseline. Significant differences are indicated by asterisks. *P<0.05.

Lumbar spine BMD was measured before surgery (baseline) and at 12 months after anticancer therapies in the premenopausal patients (Fig. 3). The OC and EC groups showed slightly but not significantly decreased lumbar spine BMD, to 94.4±4.6 and to 95.7±3.1% respectively. In other hand, the CC group who were treated with CCRT after surgical treatment showed a significant decrease in the lumbar spine BMD to 91.9±5.9% (*P*<0.05).

## Discussion

The current study demonstrated that bone resorption marker was elevated significantly 1 year after surgical menopause and adjuvant chemotherapy or CCRT in premenopausal cancer patients. The average serum BAP was not significantly elevated in the OC and EC groups; however, the power of this study to detect a significant difference was limited. These differences may be significant if a larger number of subjects were analyzed. Furthermore, surgical menopause following by CCRT induced a significant decrease in the lumbar spine BMD at 1 year after treatment in premenopausal CC patients.

There are no direct comparative longitudinal studies of natural menopause in comparison to surgical menopause on bone, so comparisons have been made between cross-sectional studies. A study on 160 women compared a group that had a natural menopause with 67 oophorectomized women, matched for age at 51 and 4 years since menopause. The authors found almost twice the rate of bone loss measured by DXA during the first 6 years in oophorectomized women in comparison to natural menopause women and a significantly higher incidence of osteopenia in the surgical group (6). Numerous studies have shown that bone loss accelerates following menopause. The earlier in life that menopause occurs, the lower the bone density will be later in life (4). Oophorectomy before age 45 is a well-established risk factor for osteoporosis (16). In addition, even the risk of osteoporotic fracture may be increased in women who undergo bilateral oophorectomy after natural menopause, in comparison to women with intact ovaries (17). Therefore, in this study surgical menopause might play a role in the bone loss in premenopausal patients after anticancer treatments.

We failed to show whether surgical castration itself affects the bone density, because only a small number of cases received bilateral oophorectomy alone without any adjuvant therapies after surgery. Some prospective studies have described a 2–2.5% annual rate of bone loss during the first 5 years after natural menopause (18, 19). Yoshida *et al*. (20) recently performed a prospective study which demonstrated the loss of BMD to be −6.7% at 12 months in women who undergo surgical menopause. The current study showed the loss of BMD to be as much as 8.1% in premenopausal CC women who received combination therapy consisting of surgical castration and CCRT. Although, this study did not show whether surgical castration, chemotherapy, or CCRT was dominantly associated with the observed lower BMD, the combination of surgical castration and CCRT clearly had a strong impact on the decreased BMD.

Several reports indicate that anticancer agents induce bone mineral loss in patients with Hodgkin disease (21, 22), breast cancer (23), and lymphoblastic leukemia (9). However, most studies have documented bone mineral loss as a symptom of the gonadal dysfunction induced by anticancer agents. There are few studies investigating the direct effect of chemotherapy on bone mineral loss. The current study demonstrated that the BMD tended to decrease, but not significantly, 1 year after surgical castration and adjuvant chemotherapy in endometrial and OC patients. Larger studies, followed over a longer time course, will be required to determine whether paclitaxel or cisplatin affects bone loss independently of hypogonadism.

There are few studies on the association between reduced BMD and CC. Hwang *et al*. (24) demonstrated that postmenopausal women with CC treated with CCRT had lower BMD and higher bone turnover marker levels than matched control women with a history of hysterectomy for uterine fibromas. Radiation has a direct effect on the bone and an indirect effect associated with vascular changes (13). Pelvic irradiation is a common predisposing factor for sacral insufficiency fractures (25). Femoral neck fractures occur in ∼2% of women who undergo pelvic radiation therapy for CC (26). On other hand, Chen *et al*. (27) showed no significant change in the BMDs (L2–L5) between women with CC treated with radiotherapy and matched controls. The current study showed that combination therapy of surgical castration and CCRT for premenopausal women with CC was associated with a significant decrease in the spine BMD. Further investigations might therefore be needed to consider premenopausal women who received surgical castration alone or ovarian conservation following by CCRT in order to evaluate the negative effect of CCRT itself.

In conclusion, premenopausal women with uterine CC treated by surgical castration and CCRT should be considered to be at risk for bone loss over the course of the subsequent year. Numerous studies have so far shown that surgical menopause itself is a risk for osteoporosis. Although our short-term data do not support routine screening except in the CCRT cohort, bone density measurement should probably be considered in all premenopausal gynecological cancer patients who receive surgical castration and adjuvant therapies. Recent prospective study demonstrated that estrogen replacement therapy reduced osteoporotic fractures in women with surgical menopause (28). Therefore, estrogen replacement therapy may prevent some degree of such bone mineral loss after anticancer treatments in premenopausal women with estrogen independent malignancies. In addition, treatment with medications, such as calcium, vitamin D, bisphosphonates, and selective estrogen receptor modulators, as well as regular exercise are also important for the prevention of fractures. Further prospective studies with a large series of cases and for longer periods of time are therefore needed to clarify the mechanisms associated with a lower BMD among women with gynecological caner treated with surgical castration following by adjuvant therapies.

## Figures and Tables

**Table 1:** Patient characteristics according to cancer type. Data are given as the mean (s.d.) unless otherwise stated.

	**OC** (*n*=15) **mean**	**EC** (*n*=9) **mean**	**CC** (*n*=11) **mean**
Age at diagnosis (years) (s.d.)	38.9 (5.8)	42.9 (6.2)	41.1 (6.6)
BMI (kg/m^2^) (s.d.)	21.7 (3.1)	23.8 (5.0)	21.5 (3.5)
Body turnover marker			
Serum BAP (U/l) (s.d.)	15.1 (4.5)	21.0 (3.1)	13.3 (4.3)
Urine NTx (nmolBCE/mmolCr) (s.d.)	25.0 (9.2)	28.0 (10.9)	23.1 (12.6)
Bone mineral density			
g/cm^2^ (s.d.)	1.076 (0.111)	1.036 (0.085)	1.149 (0.081)
*T*-score (s.d.)	−0.36 (1.44)	−0.62 (1.34)	0.33 (1.21)
Stage cancer, no. (%)			
I	9 (60)	7 (78)	9 (82)
II	1 (6)	0 (0)	2 (18)
III and IV	5 (34)	2 (22)	0 (0)

OC, ovarian cancer; EC, endometrial cancer; CC, cervical cancer; BAP, bone alkaline phosphatase; NTx, type I collagen N-terminal telopeptide; BCE, bone collagen equivalent.
